# Can Impaired Elasticity of Aorta Predict the Success of Vardenafil Treatment in Patients with Erectile Dysfunction?

**DOI:** 10.1155/2016/4867984

**Published:** 2016-04-21

**Authors:** Hüseyin Ede, Serhat Tanik, Barış Yaylak, Kürşad Zengın, Sebahattin Albayrak, Suleyman Akkaya, Cegergun Polat, Yaşar Turan, Alirıza Erbay

**Affiliations:** ^1^Department of Cardiology, Faculty of Medicine, Bozok University, 66020 Yozgat, Turkey; ^2^Department of Urology, Faculty of Medicine, Bozok University, 66020 Yozgat, Turkey; ^3^Cardiology Clinic, Diyarbakır Gazi Yaşargil Education and Research Hospital, 21010 Diyarbakır, Turkey; ^4^Department of Cardiology, Memorial Diyarbakir Hospital, 21070 Diyarbakir, Turkey

## Abstract

*Objective*. Vardenafil is used in treatment of erectile dysfunction (ED) but reveals variable clinical outcomes. Here, we aimed to evaluate the role of aortic elasticity in predicting vardenafil success among patients with ED.* Methods*. Sixty-one consecutive male subjects with primary ED and indication for vardenafil treatment were included. All subjects fulfilled 5-item version of the International Index of Erectile Function (IIEF-5) before the vardenafil treatment. Pretreatment aortic stiffness index (ASI) and aortic distensibility (AD) were obtained echocardiographically. Following two-month vardenafil treatment, the patients were reevaluated with IIEF-5. Pretreatment, posttreatment, and ΔIIEF-5 scores and ASI values were compared.* Results*. Average age was 54 ± 8 years. Pretreatment and posttreatment IIEF-5 and ΔIIEF-5 scores were 9.1 ± 2.5; 18.5 ± 2.3; and 9.4 ± 3, respectively. Mean ASI and AD values were 3.10 ± 0.54 and 4.13 ± 2.55 1/(10^3^  ×  mmHg) accordingly. ASI value of severe pretreatment ED (*n* = 15) was significantly higher than that of mild-moderate pretreatment ED (*n* = 12) (*p* < 0.001). All pretreatment IIEF-5 scores increased significantly compared to posttreatment IIEF-5 scores (*p* < 0.001). ASI values were significantly correlated to pretreatment IIEF-5 scores (*p* < 0.001) and ΔIIEF-5 value (*p* < 0.001) but not to posttreatment IIEF-5 score.* Conclusion*. Aortic elasticity was impaired in accordance with degree of ED. The subjects with higher ASI values obtained more benefits from vardenafil.

## 1. Introduction

Erectile dysfunction (ED) is common health problem of male population, currently affecting over than 152 million adults worldwide [1]. It shares similar risk factors with cardiovascular disease as aging, cigarette smoking, high blood pressure, dyslipidemia, obesity, and diabetes mellitus [2]. Thus, its prevalence increases remarkably with age. Since it is observed together with other comorbid diseases which impair the quality of life, the treatment of ED is very important to diminish additional impairment in the quality of life.

Phosphodiesterase type 5 inhibitors (PDE5Is) have been commonly prescribed for the treatment of ED. Sildenafil, tadalafil, vardenafil, and avanafil are among these PDE5Is. Unfortunately, these drugs do not show the same level of efficacy among all patients with ED and consistency rate for the prescribed drug varies significantly on individual base [3]. Until now, there has not been any guideline providing special indication for particular type of PDE5Is. Although there have been few studies searching predictors of PDE5I success, the physicians still do not have clinical parameters defining subjects who may benefit from PDE5Is [4, 5].

The reason for this uncertainty can be due to different underlying mechanisms postulated for pathophysiology of ED. Organic causes such as ischemia, smooth muscle damage or altered blood flow, androgen deficiency, performance anxiety, Alzheimer's disease, Parkinson's disease, and prescription drugs are some of them [5, 6]. Even vascular causes can be different with varying clinical implications such as arterial or venoocclusive dysfunction [5]. Additionally, considering the acting mechanisms of PDE5Is is also important to predict PDE5I success. All PDE5Is induce vasodilation via regulating vascular nitric oxide and guanylate cyclase. Thus, they are especially effective in case of ED with underlying vascular pathologies rather than psychological or neurological pathologies. Thus, it can be hypothesized that detection of underlying vascular pathology can guide the clinician to prescribe medication targeting penile vasculature such as PDE5Is.

It will be very beneficial tool if it has ability to differentiate the subjects in respect to PDE5I success. Measurement of aortic elasticity can be an option for this purpose. It reflects subclinical vascular dysfunction and predicts cardiovascular adverse events and mortality [7]. Aortic stiffness index (ASI) and aortic distensibility (AD) are parameters reflecting aortic elasticity and can be measured noninvasively by the help of echocardiography at office setting.

In this study, we assessed aortic elasticity of the patients with ED before vardenafil treatment. Efficacy of the treatment was evaluated following two-month period. Ability of aortic elasticity in predicting efficacy of the drug was tested.

## 2. Methods

### 2.1. Subjects

Patients with ED symptoms who did not get benefit from lifestyle modification and indicated for PDE5I usage were included in the study between September 2014 and May 2015. All patients were assessed with a thorough physical examination and basic neurological and psychiatric evaluations. Complete blood count, fasting blood glucose, serum lipid profile, hormonal profile with total testosterone, and prolactin and thyroid-stimulating hormone levels of each subject were obtained at the first visit. All patients were referred to cardiology department to exclude any underlying cardiovascular cause for ED. Thus, following detailed cardiological evaluation with electrocardiography and echocardiography, all subjects performed routine exercise treadmill test using the modified Bruce protocol. The patients who could not achieve at least six-minute exercise and 85% of targeted heart rate (220 minus age in years) were not included in the study. Also those with abnormal treadmill test results (severe angina, sustained ventricular arrhythmia, exertional hypotension, and ST depression of more than 1 mm at two consecutive leads) were excluded from the study.

The other exclusion criteria for the study were as follows: patients with underlying surgical reason for ED (congenital penile curvature, Peyronie's disease, and hypospadias); presence of neurologic or psychiatric disorders; presence of hormonal disorders; previous PDE5I usage; current use of medications which may lead ED such as diuretics, beta-blockers, antiandrogens, antidepressants, and antipsychotics; presence of known obstructive coronary artery disease; congestive heart failure; left ventricle ejection fraction (LVEF) of <50%; documented acute coronary syndrome within the last six months; presence of moderate or severe valvular heart disease; presence of hypertrophic cardiomyopathy; pulmonary hypertension (average pulmonary artery systolic pressure >30 mmHg); presence of prolonged corrected QT interval (>460 msn); diabetes mellitus; uncontrolled hypertension; malignancy; renal failure [glomerular filtration rate (GFR) of <60 mL/min/1.73 m^2^ calculated using GFR = 0.55 × height in cm/serum creatinine in mg/dL]; patients with transaminase levels of more than two times the upper limit of normal level; patients under nitrate treatment; and patients who used selective PDE5Is within the last three months. Erectile dysfunction severity of the patients without exclusion criteria was evaluated by the help of an abridged 5-item version of the International Index of Erectile Function (IIEF-5) at the beginning of the study. Then, the patients were advised to use 10 mg vardenafil tablet before sexual intercourses for two months (one tablet for each sexual intercourse and use of at least 8 pills within this duration). The patients who used at least 8 ten-milligram vardenafil pills within this duration (maximum 12 pills) were reevaluated in respect to ED severity by the help of IIEF-5 at the end of two-month vardenafil treatment [4]. The difference between the pretreatment and posttreatment IIEF-5 values was accepted as ΔIIEF-5. The severity level of ED in respect to IIEF-5 scores was defined in accordance with the description of Rosen et al. [8].

The Local Ethical Committee has approved the study and informed consent of each patient was provided. The study was performed according to rules set by the Declaration of Helsinki.

### 2.2. Assessment of Aortic Elasticity

Detailed echocardiographic examination was performed on an ultrasound machine (ProSound Alpha 7, IPF 1701 Model, 2009; Hitachi Aloka Medical, Ltd., Tokyo, Japan) with a 2.5 MHz transducer by cardiologist blinded to the patient's data. Standard two-dimensional measurements (left ventricle diastolic and systolic diameters, ventricular septal and posterior wall thickness, left atrial diameter, and LVEF) were obtained as recommended by the American Society of Echocardiography [9]. The mitral inflow velocities were traced and peak velocity of early diastolic mitral inflow (*E*) and late diastolic mitral inflow (*A*) were acquired. The left ventricle lateral wall basal segment tissue velocities were gained by tissue Doppler imaging (TDI) via the pulsed-wave mode. The filter was set to exclude high-frequency signals, and the Nyquist limit was arranged to a range of 15 to 20 cm/s. Gain and sample volume were decreased as much as possible to acquire a clear tissue signal with minimal background noise. Early diastolic (*E*′), late diastolic (*A*′), and systolic velocities (*S*) of the left ventricle lateral wall basal segment tissue were obtained from the apical 4-chamber view with a 2 to 5 mm sample volume placed at the left ventricle lateral wall basal segment. All measurements were performed at the end of expirium. Normal diastolic function was defined as *E*/*A* ratio > 1, *E*′/*A*′ > 1, and *E*/*E* m < 8; Grade I diastolic dysfunction (DD) was defined as *E*/*A* ratio < 1, *E*′/*A*′ < 1, and *E*/*E*′ < 8; Grade II DD was defined as *E*/*A* ratio > 1 and < 2, *E*′/*A*′ < 1, and *E*/*E*′ between 8 and 15.

The blood pressure (BP) levels were obtained from the right and left arms of the patients in a sitting position by one experienced observer blinded to clinical data of the patients in the echocardiography laboratory. BP was acquired twice within five-minute interval. The systolic BP (SBP) and diastolic BP (DBP) were recorded at the first and fifth Korotkoff phases accordingly using a mercury sphygmomanometer. The average of four BP values was used for analysis. The difference of SBP and DBP was used as pulse pressure (PP).

After the echocardiographic evaluation, at parasternal long axis M-mode images, the systolic (Asd) and diastolic (Add) aortic diameters of ascending aorta from lower margin of upper wall to upper margin of lower wall were obtained 3 cm distal to the aortic valve level, differentiating diastole and systole by using contemporaneous ECG recordings. While aortic stiffness index is obtained by the formula ASI = ln⁡(SBP/DBP)/[(Asd − Add)/Add], aortic distensibility is calculated by using AD [1/(10^3^  ×  mmHg)] = 2 × [(Asd − Add)/Add]/PP formula [10].

### 2.3. Statistical Analysis

Statistical analyses were carried out by using the SPSS software version 18. Continuous variables are expressed in mean ± SD and categorical variables are shown as frequencies (%). Except TDI *E*′/*A*′ ratio and *E*/*E*′ ratio all other continuous variables had normal distribution according to Kolmogorov-Smirnov test. Categorical variables were analysed by using Pearson chi-square test. Spearman correlation analysis was used to search association between TDI *E*′/*A*′ and *E*/*E*′ ratio and IIEF-5 scores while Pearson simple correlation analyses were applied to search the association between IIEF-5, laboratory, and the other echocardiographic parameters. Student's *t*-test and ANOVA were used to test the difference between the groups in respect to continuous variables except TDI *E*′/*A*′ and *E*/*E*′ ratios in which Mann-Whitney *U* and Kruskal-Wallis tests were used accordingly. Paired-samples *t*-test was used to compare the pretreatment and posttreatment IIEF-5 values. The multiple linear regression analysis was carried out to detect independent association between ΔIIEF-5 and potential confounding variables with normal distribution and significant correlation (age, total cholesterol, BMI, and ASI). A *p* value of <0.05 was considered to be statistically significant result.

## 3. Results

Seventy-one patients with primary ED were started on vardenafil treatment. Ten patients did not complete the scheduled vardenafil treatment due to side effects such as headache, nasal congestion, and flushing or they did not admit to the outpatient clinic at the end of two-month follow-up. Thus, the study included sixty-one patients with primary ED patients. Frequency of mild-moderate hypertensive patients under angiotensin converting enzyme inhibitor/angiotensin receptor blocker and/or calcium channel blockers was 21% (*n* = 13).

Average age of the patients was 53.9 ± 7.6 years. Clinical data of the subjects were summarized in Table 1. Average pretreatment IIEF-5 score of the subjects was 9.1 ± 2.5 while posttreatment score was 18.5 ± 2.3. Pretreatment IIEF-5 scores of the subjects were increased significantly following the treatment (*p* < 0.001; *z* score = −6.804). 15 patients had severe pretreatment ED, 34 had moderate pretreatment ED and 12 mild-moderate pretreatment ED while 2 moderate, 8 mild-moderate, 47 mild, and 4 without ED patients were observed at the end of two-month vardenafil treatment. Two subjects did not show any change in their own ED category following vardenafil treatment while the rest of the subjects showed at least one level of improvement in ED. There was not any statistically significant difference between the severe, moderate, and mild-moderate pretreatment ED groups in respect to the number of ten-milligram vardenafil pills used within two-month duration (9.5 ± 1.3, 9.1 ± 1.1, and 9.2 ± 1.3 pills, resp.; *p* = 0.436).

Laboratory and echocardiographic examination results of the subjects were shown in Table 1. Average LVEF and *E*/*E*′ values of the subjects were 63 ± 3% and 6.2 ± 1.8, respectively. Average ASI value was 3.10 ± 0.54 while average AD value was 4.13 ± 2.55 [1/(10^3^  ×  mmHg)].

In the correlation analyses, we found that only serum total testosterone level was significantly correlated to pretreatment IIEF-5 (*r* = 0.558, *p* < 0.001) and ΔIIEF-5 (*r* = −0.487, *p* < 0.001) scores opposite to other laboratory findings (hemoglobin, fasting blood glucose, high density lipoprotein, low density lipoprotein, triglyceride, total cholesterol, serum prolactin, and thyroid-stimulating hormone levels).

Among echocardiographic variables (LVEF, left atrial diameter, mitral inflow *E*/*A* ratio, and TDI *E*′/*A*′ and *E*/*E*′ ratio), only TDI *E*′/*A*′ ratio showed significant correlation with pretreatment IIEF-5 (*r* = 0.480, *p* < 0.001) and ΔIIEF-5 (*r* = −0.391, *p* < 0.005) scores, respectively. 22 patients (36%) had normal diastolic function while there were 30 patients (49%) with Grade I DD and 9 patients (15%) with Grade II DD. Patients with normal diastolic function had significantly higher pretreatment IIEF-5 score compared to the patients with DD of any grade (10.9 ± 2.0 versus 8.1 ± 2.2, *p* < 0.001). Similarly, average ΔIIEF-5 score was lower among patients with normal diastolic function compared to the subjects with any degree of diastolic dysfunction (7.9 ± 2.4 versus 10.3 ± 2.9, *p* < 0.005).

Aortic elasticity was measured by using ASI and AD. ASI and AD were inversely correlated (*r* = −0.979, *p* < 0.001). ASI was negatively related to pretreatment IIEF-5 score (*r* = −0.850, *p* < 0.001) and positively correlated to ΔIIEF-5 score (*r* = 0.732, *p* < 0.001) while AD significantly associated with pretreatment IIEF-5 score (*r* = 0.834, *p* < 0.001) and was inversely correlated to ΔIIEF-5 score (*r* = −0.734, *p* < 0.001). Among the other clinical, laboratory, and echocardiographic parameters, only age, total testosterone, and TDI *E*′/*A*′ ratio were significantly correlated to pretreatment IIEF-5 scores (Table 2). Although age showed significant correlation with pretreatment IIEF-5 score, it did not have significant relation with either posttreatment IIEF-5 or ΔIIEF-5 scores.

We found that patients with severe pretreatment ED had higher ASI values compared to the patients with moderate pretreatment ED (*p* < 0.001) and mild-moderate pretreatment ED (*p* < 0.001). Similarly, the patients with moderate pretreatment ED had significantly higher ASI values compared to the patients with mild-moderate pretreatment ED (*p* < 0.001). The relation of pretreatment erectile dysfunction severity with aortic elasticity was shown in Table 3.

In multiple linear regression analysis in which ΔIIEF-5 score was taken as a dependent variable and age, BMI, total testosterone, and ASI were taken as independent variables; we found that only ASI had an independent correlation with increased ΔIIEF-5 (Table 4).

Ten patients were selected on random basis for echocardiographic ASI measurement repeated one week later to assess intraobserver variability. Reproducibility of the measurement was significantly correlated for intraobserver agreement (intraclass correlation coefficient = 0.926, *p* < 0.001).

## 4. Discussion

The knowledge about predictors of erectile dysfunction treatment is limited in the literature. In this study, we found that primary ED patients who got benefit from vardenafil treatment were the ones with higher ASI, lower AD, worse diastolic dysfunction, and lower serum total testosterone levels. Since impaired aortic elasticity is related to presence of underlying cardiovascular pathology, vardenafil success in the treatment of ED seems to be associated with penile arterial vasculature.

Erectile dysfunction is a common sexual health problem leading reduction in quality of life among males [11]. Since ED has several underlying pathophysiologic mechanisms [5, 6], it is not easy to treat. Thus, discontinuation of the prescribed treatment is common and reported to be up to 45% with sildenafil treatment. Aging, incontinence, concomitant drug use, psychogenic cause, and religion were found to be potential causes of drug discontinuation [3, 12]. All these facts imply that compliance with the treatment is multifactorial and differentiation of the patients who may benefit from prescribed treatment is essential for higher rate of compliance. In this study, discontinuation rate was 14.1%. We also designed this study to define clinical determinators helping to detect the subjects with recovery potential following vardenafil treatment.

For this purpose, measurement of aortic elasticity can be logical approach. Aging, hypertension, diabetes mellitus, atherosclerosis, and chronic renal failure are some causes of impaired aortic elasticity [13]. Thus, impaired aortic elasticity is closely related to extent of cardiovascular impairment [14]. As described before, severity of ED shares similar risk factors with cardiovascular disease and its prevalence increases remarkably among patients with risk factors for cardiovascular disease [2, 15]. Therefore, it should be expected that detection of impaired aortic elasticity in patients with primary ED also gives evidence for dysfunction of penile arterial vasculature. In our study, we also found that increased ASI was significantly correlated with pretreatment IIEF-5 and also the patients with severe pretreatment ED had more deteriorated aortic elasticity compared to the patients with moderate or mild-moderate pretreatment ED.

Pulsed-wave velocity (PWV) method has been used more frequently in the studies involving arterial stiffness rather than echocardiographic method [16, 17]. However PWV method has some limitations. Need of high-level experience and groin exposure are some limitations for its applicability [16, 17]. But it can be assessed with echocardiography as in this study. It is also reliable and reproducible and frequently used in the literature [10, 18]. As known, aortic elasticity is mostly related to changes in extracellular matrix rather than muscular layer of the vessel [19]. However, the stiffness of aorta, which is an elastic central artery, is measured directly with echocardiographic method without limitations mentioned before. Thus, we used echocardiography to determine ASI and AD. However it has also some limitations such as presence of suboptimal images, need of longer time, and possibility of interobserver variability.

Phosphodiesterase type 5 inhibitors have been commonly used in the treatment of ED. Phosphodiesterase type 5 isoenzymes have important role in nitric oxide/3′,5′-guanosine monophosphate (cGMP) signalling by transforming cGMP to its inactive form [20]. PDE5Is inhibit these enzymes and increase intracellular nitric oxide levels leading smooth muscle relaxation and vasodilation. As a result, penile erection is enabled. Any failure at this cascade will lead to ED. Thus, vardenafil improves ED due to vascular dysfunction via increasing vascular nitric oxide. Therefore, it can be concluded that the patients with higher vascular dysfunction benefit more from vardenafil treatment. In our study, we also found that the patients with higher ASI had more improvement in posttreatment IIEF-5 score and ASI was significantly correlated with ΔIIEF-5. Increased stiffness may be especially important to define the patients with high probability to benefit from vardenafil treatment. This finding was also confirmed with multiple regression analysis since only ASI showed an independent relation with increased ΔIIEF-5 score.

Vardenafil can improve aortic stiffness in acute setting within 3 hours of intake [21]. In our study, the measurements were performed at least 12 hours after intake of the last vardenafil pill. Thus, there was no significant correlation between the number of pills used and the treatment effect in our study (ΔIIEF-5 score) (*r* = −0.027, *p* = 0.835). This result can also be explained that the treatment was not given for long-term and limited to eight to twelve pills in two-month duration. Long-term continuous treatment can give different results and needs further investigation.

Relation of vardenafil and aortic elasticity among patients with ED was tested in two different studies. However, they searched different aspect of vardenafil and aortic elasticity. One of them searched acute effect of vardenafil on aortic elasticity and they measured aortic elasticity with PWV method and they found improvement in aortic elasticity 3 hours after ingestion of vardenafil [21]. This confirms the current knowledge about PDE5Is in respect to their vasodilation effect and supports our hypothesis implying that the patients with impaired ASI get more benefit from vardenafil treatment. The other study included less number of subjects with ED (*n* = 20) without grading ED severity [22]. Additionally augmentation index calculated by fingertip peripheral arterial tonometry was used to measure arterial stiffness. They found that the patients who received vardenafil 10 mg daily showed more improvement in arterial stiffness following 4-week treatment compared to the patients who received vardenafil 20 mg on demand and both groups showed better IIEF-5 scores at the end of 4-week interval [22]. However they did not conclude that subjects with higher degree of impairment in aortic stiffness obtained better outcome with vardenafil treatment. Rather, they just confirmed that four-week treatment of vardenafil 10 mg daily improved aortic stiffness among subjects with ED. However we found that severity of ED was directly correlated with aortic stiffness and only ASI was the predictor of vardenafil success measured with pre- and posttreatment IIEF-5 scores difference among patients with ED.

## 5. Limitations of the Study

The number of subjects included in the study was small. The use of vardenafil tablets could not be confirmed objectively; thus the personal claim was accepted to confirm drug continuation. Additionally, two-month treatment duration may not be long enough to assess vardenafil efficacy. Another important concern can be the measurement method of aortic elasticity, which was not obtained with pulsed-wave velocity technique.

## 6. Conclusion

Echocardiographic measurement of ASI and AD may be useful approach to define the subjects with primary ED who get more benefit from vardenafil treatment.

## Figures and Tables

**Table 1:** Clinical, laboratory, and echocardiographic findings of the subjects.

*n* = 61	Mean ± standard deviation
Age, year	53.9 ± 7.6
Weight, kg	83.1 ± 13.3
Height, m	1.71 ± 0.07
BMI, kg/m^2^	28.4 ± 4.3
SBP, mmHg	119 ± 13
DBP, mmHg	77 ± 6
Pretreatment IIEF-5 score	9.1 ± 2.5
Posttreatment IIEF-5 score	18.5 ± 2.3
ΔIIEF-5 score	9.4 ± 3.0
FBG, mg/dL	103 ± 12
Total cholesterol, mg/dL	199 ± 32
Triglyceride, mg/dL	185 ± 120
HDL, mg/dL	41 ± 8
LDL, mg/dL	126 ± 25
TSH, *μ*IU/mL	1.43 ± 0.88
Prolactin, ng/mL	9.0 ± 4.6
Total testosterone, mg/dL	580 ± 128
Hgb, gr/dL	14.6 ± 0.8
LVEF, %	63 ± 3
LA diameter, cm	3.9 ± 0.3
Mitral *E*/*A* ratio	1.07 ± 0.47
TDI *E*′/*A*′ ratio	1.06 ± 0.58
*E*/*E*′ ratio	6.2 ± 1.8
ASI	3.10 ± 0.54
AD, 1/(10^3^ × mmHg)	4.13 ± 2.55
Number of pills used within two months	9.2 ± 1.2

SBP: systolic blood pressure; DBP: diastolic blood pressure; BMI: body mass index; IIEF-5: International Index of Erectile Function; LDL: Low-density lipoprotein; HDL: high-density lipoprotein; TSH: thyroid-stimulating hormone; FBG: fasting blood glucose; Hgb: hemoglobin; LVEF: left ventricle ejection fraction; LA diameter: left atrium diameter; TDI *E*′/*A*′: the left ventricle lateral wall basal segment tissue Doppler imaging *E*′/*A*′ ratio; *E*/*E*′ ratio: mitral inflow *E*/the left ventricle lateral wall basal segment tissue Doppler imaging *E*′ ratio; ASI: aortic stiffness index; AD: aortic distensibility.

**Table 2:** Correlation analyses results of the subjects' data and IIEF-5 scores were expressed.

	Pretreatment IIEF-5 score	Posttreatment IIEF-5 score	ΔIIEF-5 score
Age, year	*r* = −0.356, *p* < 0.01	*r* = −0.056, *p* = 0.666	*r* = 0.211, *p* < 0.103
BMI, kg/m^2^	*r* = −0.022, *p* = 0.864	*r* = 0.228, *p* = 0.077	*r* = 0.176, *p* = 0.174
Total testosterone, mg/dL	*r* = 0.558, *p* < 0.001	*r* = −0.078, *p* = 0.550	*r* = −0.487, *p* < 0.001
TDI *E*′/*A*′ ratio	*r* = 0.480, *p* < 0.001	*r* = −0.012, *p* = 0.930	*r* = −0.391, *p* < 0.005
ASI	*r* = −0.850, *p* < 0.001	*r* = 0.023, *p* < 0.860	*r* = 0.732, *p* < 0.001
AD, 1/(10^3^ × mmHg)	*r* = 0.834, *p* < 0.001	*r* = −0.049, *p* < 0.708	*r* = −0.734, *p* < 0.001

BMI: body mass index; ASI: aortic stiffness index; AD: aortic distensibility; IIEF-5: International Index of Erectile Function; TDI *E*′/*A*′ ratio: the left ventricle lateral wall basal segment tissue Doppler imaging *E*′/*A*′ ratio.

**Table 3:** The relation of pretreatment erectile dysfunction severity with aortic elasticity.

	Severe ED (*n* = 15)	Moderate ED (*n* = 34)	Mild-moderate ED (*n* = 12)	*p* value
ASI	3.68 ± 0.33	3.06 ± 0.43	2.49 ± 0.22	<0.001
AD, 1/(10^3^ × mmHg)	1.82 ± 0.69	4.01 ± 2.13	7.35 ± 1.61	<0.001

ASI: aortic stiffness index; AD: aortic distensibility; ED: erectile dysfunction.

**Table 4:** Independent association between ΔIIEF-5 and the study parameters by multiple linear regression analysis was shown (*r* ^2^ = 0.511, *p* < 0.001).

	Beta regression coefficient	*p* value
Age, year	−0.128	0.253
Total testosterone, mg/dL	−0.157	0.209
BMI, kg/m^2^	0.139	0.155
ASI	0.620	<0.001

ASI: aortic stiffness index; BMI: body mass index; IIEF-5: International Index of Erectile Function.
